# Glandular tissue–based versus anatomical landmark–based CTV delineation in postoperative breast cancer radiotherapy: a dosimetric comparison

**DOI:** 10.3389/fonc.2026.1825988

**Published:** 2026-05-29

**Authors:** Chao Li, Bin Zhang, Lin Huang, Qiu-Ming Wei, Su-Fang Qiu

**Affiliations:** 1 College of Clinical Medicine for Oncology, Fujian Medical University, Fuzhou, China; 2 Department of Oncology, Second Hospital of Sanming City, Sanming, China; 3 Department of Radiation Oncology, Clinical Oncology School of Fujian Medical University, Fujian Cancer Hospital, NHC Key Laboratory of Cancer Metabolism, Fuzhou, China

**Keywords:** breast cancer, cardiac protection, CTV, dosimetric analysis, OARs, postoperative radiotherapy, radiation-induced toxicity

## Abstract

**Background:**

Precise delineation of clinical target volume (CTV) in postoperative breast cancer radiotherapy to ensure adequate target coverage while limiting radiation exposure to organs at risk (OARs). Anatomical landmark–based and glandular tissue–based approaches are widely used, but their dosimetric differences and practical implications remain under discussion.

**Methods:**

This retrospective dosimetric study included breast cancer patients who underwent breast-conserving surgery with normal bilateral breast morphology. CT simulations (3-mm slice thickness) were imported into the Monaco 5.0 planning system. Two CTV delineation strategies were compared: anatomical landmark–based (CTV*_an_*) following ESTRO guidelines, and glandular tissue–based (CTV*_gl_*) using CT visualization. Planning target volume (PTV) was generated by applying a 0.5-cm retraction from the skin and a 0.5-cm uniform expansion. All contours were generated by a single senior radiation oncologist, and treatment plans were created by an experienced physicist. Dosimetric parameters for the heart, left lung, and contralateral breast were evaluated.

**Results:**

A total of 48 patients (age 32–67 years; mean 49.35 ± 8.57) were included. CTV*_gl_* volumes were significantly smaller than CTV*_an_* volumes (432.87 ± 184.09 cc vs. 582.59 ± 232.55 cc; *P* < 0.001). Glandular-based delineation was associated with lower D*_max_*, higher D_98_, and reduced OARs exposure. Left lung V_20_, mean heart dose, heart V_40_, and contralateral breast(CB) D*_mean_* were all significantly lower in the CTV*_gl_* group (*P* < 0.001). CTV volume showed significant positive associations with heart and contralateral-breast dose, whereas left-lung dose demonstrated weaker and more variable associations, reflecting the influence of anatomical and planning-related factors.

**Conclusions:**

Glandular tissue–based delineation produced smaller target volumes and lower radiation exposure to adjacent organs compared with anatomical landmark–based delineation. These findings provide quantitative dosimetric evidence of differences between the two approaches; however, the clinical implications of these reductions cannot be inferred from this dosimetric analysis alone. Further studies incorporating normal tissue complication probability (NTCP) modeling and clinical outcomes are needed to determine their relevance for long-term toxicity and treatment decision-making.

## Introduce

Breast cancer is the most common malignant tumor in women, and its incidence has continued to rise in recent years (1). Improvements in screening have facilitated earlier detection, and breast-conserving surgery has become the preferred option for many patients with early-stage disease due to favorable cosmetic and oncologic outcomes. As survival improves, postoperative radiotherapy is now a central component of treatment. As survival rates improve, attention has increasingly shifted toward long-term treatment-related toxicities, particularly radiation-induced cardiac and pulmonary injury. The close anatomical proximity of the breast to the heart and lungs—especially in left-sided tumors—makes incidental irradiation difficult to avoid, prompting extensive research into strategies that minimize exposure to OARs.

Modern radiotherapy technologies and delivery techniques have enhanced the ability to spare OARs (2–4). However, optimal protection begins with accurate and individualized target delineation. Breast anatomy varies considerably among women, particularly with respect to glandular tissue volume and distribution. Current ESTRO guidelines endorse two acceptable approaches to breast target delineation: one based on predefined anatomical landmarks and the other based on mammary gland tissue visible on CT (5, 6). The relative advantages of these approaches remain debated. Anatomical-landmark delineation was historically favored because gland margins can be difficult to visualize on CT (7), although subsequent studies have shown that CT and MRI yield comparable glandular volumes and that advances in imaging and positioning have improved gland visibility (8).

Because radiotherapy can cause permanent cardiac damage (9), unnecessarily large CTVs generated by anatomical-landmark delineation may increase radiation exposure to adjacent organs. To better understand the dosimetric implications of these two delineation strategies, we compared heart and lung doses resulting from glandular tissue–based versus anatomical landmark–based CTV definitions in patients undergoing postoperative breast radiotherapy.

## Patient selection and imaging protocol

All patients included in this study had undergone breast-conserving surgery and received postoperative radiotherapy at our oncology center. Eligible patients exhibited normal bilateral breast morphology aside from expected postoperative changes, and indications for adjuvant radiotherapy indications were determined according to institutional guidelines. Patients with incomplete imaging data or a history of prior thoracic radiotherapy were excluded.

CT simulations were performed using a GE Revolution scanner with a slice thickness of 3 mm, covering the region from the upper neck to 10 cm below the inframammary fold. The acquired datasets were imported into the Monaco 5.0 treatment planning system (Elekta AB, Stockholm, Sweden) for dosimetric evaluation.

## Target and organ-at-risk delineation

**Figure 1:**
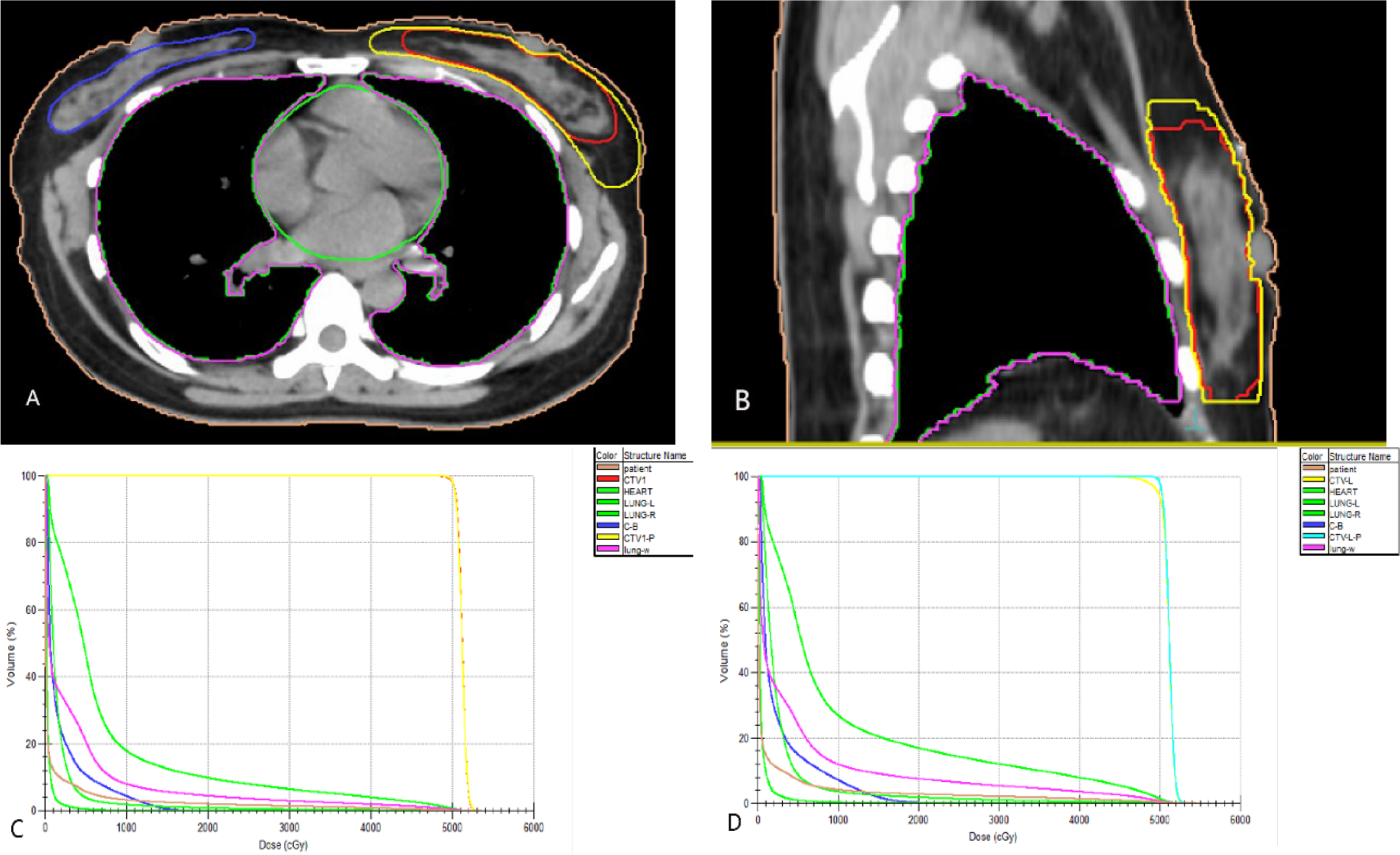
**(A, B)** The red curve depicts the CTV delineated according to mammary gland structures, while the yellow curve represents the CTV delineated using anatomical landmarks. **(C)** DVH curve corresponding to the treatment plan based on the CTV*_gl_* target volume. **(D)** DVH curve corresponding to the treatment plan based on the CTV*_an_* target volume.

During IMRT simulation, patients were immobilized in the supine position using a vacuum pad and thermal mold, with both arms positioned overhead to enhance reproducibility and treatment stability. Each patient underwent two CTV delineation approaches for the left breast: CTV*_an_*, defined according to anatomical landmarks recommended by the ESTRO guidelines, and CTV*_gl_*, derived from direct visualization of mammary gland structures on CT imaging (window width 300, window level 0) (**Figures 1A, B**).

The planning target volume (PTV) was generated by applying a 0.5 cm retraction from the skin surface and a uniform 0.5 cm expansion in all other directions. All contours were created by a senior radiation oncologist with more than 20 years of clinical experience, and treatment plans were developed by an experienced medical physicist. Two IMRT plans—one for each CTV definition—were generated for every patient to enable paired dosimetric comparison.

All treatment plans were optimized using 6-MV photon beams. The prescribed dose was 50 Gy delivered in 25 fractions, with planning objectives ensuring that at least 95% of the PTV receive 100% of the prescription dose. Dose variations within the target were constrained to ±5% in accordance with standard radiation physics requirements. **Figures 1C, D** presents representative dose–volume histogram (DVHs) illustrating the dosimetric differences between the two delineation strategies. 

## Organs at risk

Organs at risk were delineated according to ESTRO and institutional contouring guidelines. The following structures were contoured for dosimetric evaluation:

- Heart: delineated from the pulmonary trunk superiorly to the apex inferiorly, excluding major vessels.
- Left Lung: contoured in its entirety, excluding the trachea and main bronchi.
- CB: delineated as the visible breast tissue on the right breast. Dose constraints were applied in accordance with international recommendations:
- Heart: mean dose (D*_mean_*) and maximum dose (D*_max_*) were recorded, with V_40_ used to assess high-dose exposure.
- Left Lung: D*_max_*,D*_mean_*, V_20_, and V_5_ were evaluated to quantify pulmonary dose burden.
- Contralateral Breast: D*_mean_*, V_10,_ V_5,_ and D*_max_* were measured to assess scatter and unintended exposure.

## Observation criteria

- Comparison of target volumes and radiation dose distributions between CTV*_gl_* and CTV*_an_*.
- Correlation between CTV volume and radiation exposure to OARs.

## Statistical analysis

All statistical analyses were performed using SPSS (version 22, IBM Corp., Armonk, NY, USA). Continuous variables were expressed as mean ± standard deviation (SD), and categorical variables as frequencies and percentages. Dosimetric parameters for OARs - including the heart, left lung, and CB - were compared between the CTV*_gl_* and CTV*_an_* plans using paired t-tests, reflecting the within-patient paired study design.

Correlation analyses were conducted to examine the association between CTV volume and radiation dose parameters (D*_max_*, D*_mean_*, V_40_). Spearman’s rank correlation coefficients were calculated to assess the strength and direction of associations. For parameters demonstrating statistically significant correlations, simple linear regression was performed to further characterize the relationship between CTV volume and organ dose. Because all dosimetric parameters were derived from the same cohort of patients, the correlation analyses involved within-subject dependency. Accordingly, these analyses were interpreted as exploratory assessments of association rather than inferential tests based on independent observations.

A two-tailed *P* value < 0.05 was considered statistically significant. No formal adjustment for multiple comparisons was applied; therefore, the risk of type I error should be acknowledged when interpreting the results. All analyses were independently verified to ensure reproducibility and accuracy.

This was a retrospective dosimetric study; therefore, no *a priori* sample size calculation was performed. A *post hoc* power analysis based on the paired difference in CTV volume between the CTV*_gl_* and CTV*_an_* plans demonstrated a large effect size (Cohen’s d_z > 1.3). With 48 paired cases and a two-sided *α* = 0.05, the statistical power exceeded 99%, indicating that the available sample size was sufficient to detect the primary dosimetric differences between the two delineation strategies.

## Result

A total of 48 patients (aged 32–67 years; mean 49.35 ± 8.57) who underwent breast-conserving surgery for breast cancer between January 2023 and October 2025 were enrolled at the Department of Oncology, Second Hospital of Sanming City (**Table 1**). In treatment planning, the homogeneity index (HI) and conformity index (CI) are ideally close to 1. In this study, CI values for the CTV*_gl_* and CTV*_an_* groups were 0.816 ± 0.039 and 0.824 ± 0.031, respectively (*P* = 0.021), while HI values were 1.036 ± 0.01 and 1.040 ± 0.01 (*P* < 0.001). D_98_ values were higher in the CTV*_gl_* group (4981.77 ± 60.98 vs. 4954.36 ± 66.34; *P* < 0.001). The maximum point dose within the target was lower in the CTV*_gl_* group, whereas the minimum point dose was higher (**Table 2**). Although the differences in CI and HI reached statistical significance, the absolute magnitude of these differences was small and unlikely to be clinically meaningful.

**Table 1:** Patient characteristics.

Characteristic	Value
Number of patients	48
Age (years), mean ± SD	49.35 ± 8.57
Age range (years)	32–67
BMI (kg/m²), mean ± SD	24.4 ± 3.36
T stage, n (%)	Tis:2(4.2%),T1:28(58.3%),T2:18(37.5%)
N stage, n (%)	N0:34(70.8%), N1:13(27.1%), N2:1(2.1%)
Surgery type	Breast-conserving surgery
Breast side treated	Left breast (all patients)
Breast morphology	Normal bilateral morphology (post-surgical changes only)
Radiotherapy technique	IMRT, 6 MV

**Figure 2:**
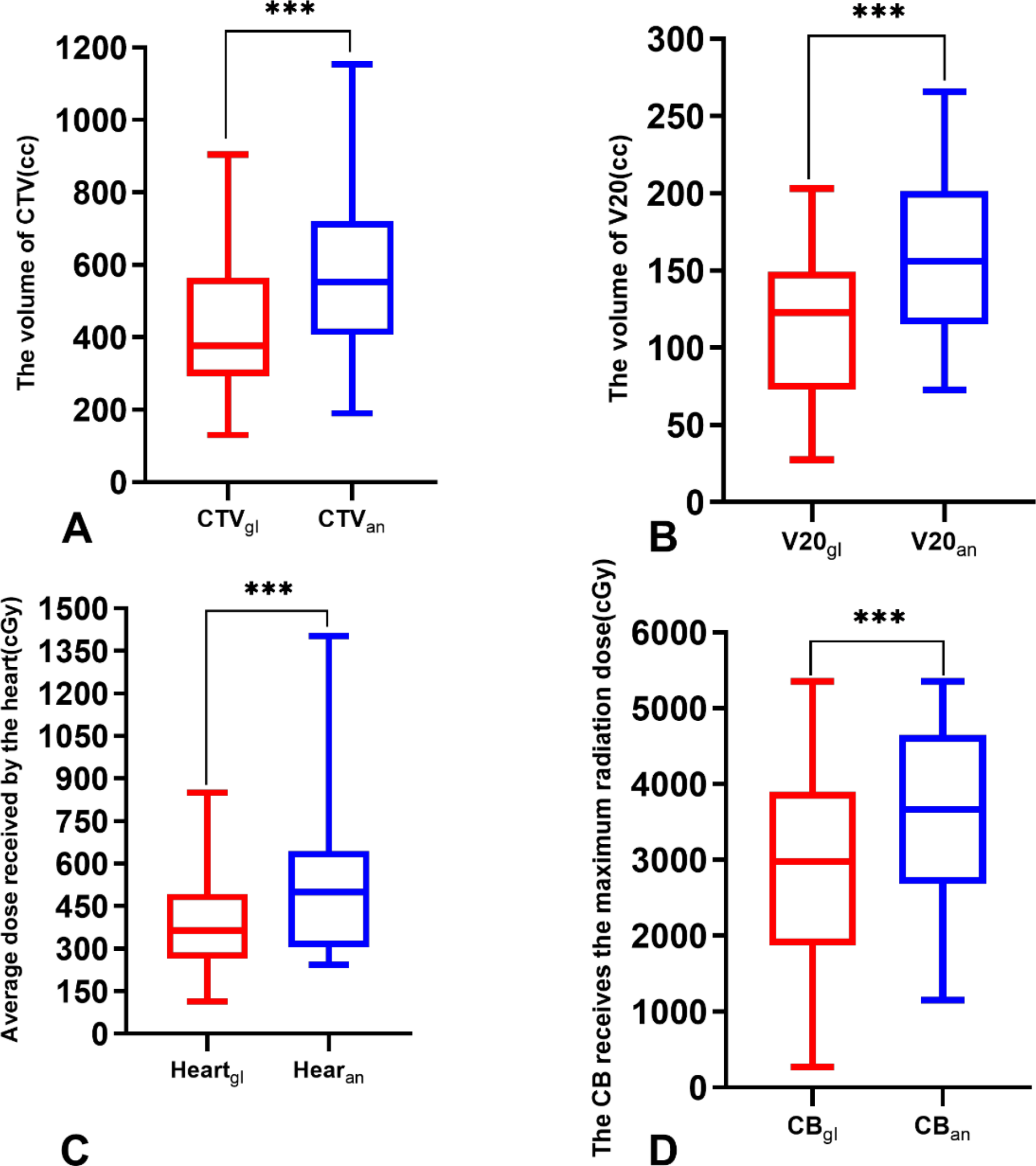
Box plot comparisons of tissue and organ volumes across different clinical groupings. **(A)** Differences in CTV volumes between glandular tissue–based (CTV*_gl_*) and anatomical landmark–based (CTV*_an_*) contouring methods. **(B)** Comparison of V_20_ volumes between CTV*_gl_* and CTV*_an_* groups. **(C)** Variation in mean heart dose (Heart*_mean_*) between CTV*_gl_* and CTV*_an_* groups. **(D)** Differences in maximum contralateral breast dose (CB*_max_*) between CTV*_gl_* and CTV*_an_* groups. ***, P < 0.001.

CTV volumes differed significantly between the two delineation methods, with CTV*_gl_* measuring 432.87 ± 184.09 cc compared to 582.59 ± 232.55 cc for CTV*_an_* (*P* < 0.001). Dosimetric comparisons demonstrated that gland-based delineation consistently reduced radiation exposure to organs at risk. The volumes of V_20_, Heart*_mean_*, and CB*_max_* dose were all significantly lower in the CTV*_gl_* group (*P* < 0.001) (**Figures 2**). Significant differences were also observed in both maximum and minimum values of left lung and heart dose. Overall, radiation exposure to the left lung, heart, and contralateral breast was markedly reduced in the CTV*_gl_* group, with parameters including left lung V_20_, heart D*_mean_*, heart V_40_, and contralateral breast D*_mean_* consistently lower. Other organ-related dosimetric indices likewise showed significant reductions (**Table 2**).

**Table 2:** Comparative assessment of treatment plans using two different delineation approaches.

Parameter	CTV*_gl_* group (mean ± SD)	CTV*_an_* group (mean ± SD)	t	*P*
CI	0.816 ± 0.04	0.824 ± 0.03	-2.38	*0.021*
HI	1.036 ± 0.01	1.040 ± 0.01	-4.463	<0.001
D_98_	4981.77 ± 60.98	4954.36 ± 66.34	7.121	<0.001
D*_max_*	5385.28 ± 41.31	5407.01 ± 71.20	-2.566	0.014
D*_min_*	4289.18 ± 472.86	4130.09 ± 507.45	4.441	<0.001
CTV _(cc)_	432.87 ± 184.09	582.59 ± 232.55	-9.434	<0.001
Heart *_max_*_(cGy)_	4721.15 ± 807.96	5003.80 ± 428.42	-3.224	0.002
Heart *_mean_*_(cGy)_	384.69 ± 176.73	503.71 ± 220.07	-5.498	<0.001
Heartv_40(cc)_	4.35 ± 7.13	10.32 ± 14.20	-4.473	<0.001
Lung-L *_max_*	5244.26 ± 120.10	5298.72 ± 120.10	-3.402	0.001
Lung-L *_mean_*	839.66 ± 172.46	1015.31 ± 142.15	-9.667	<0.001
Lung-Lv_20(cc)_	111.03 ± 46.82	156.17 ± 52.11	-10.391	<0.001
Lung-Lv_20(%)_	11.03 ± 3.94	15.50 ± 3.42	-11.436	<0.001
Lung-Lv_5(cc)_	493.64 ± 168.35	517.30 ± 153.59	-2.027	0.048
Lung-Lv_5(%)_	49.31 ± 11.46	51.90 ± 10.08	-2.218	0.031
CB*_max_*_(cGy)_	2976.01 ± 1234.07	3558.34 ± 1159.84	-5.961	<0.001
CB *_mean_*_(cGy)_	436.26 ± 289.23	550.90 ± 308.85	-5.712	<0.001
CBv_10(cc)_	37.37 ± 52.14	48.58 ± 61.13	-3.26	0.002
CBv_5(cc)_	55.24 ± 60.03	68.14 ± 73.70	-3.646	<0.001

Correlation analysis revealed a strong positive association between CTV volume and radiation doses to the heart and contralateral breast, whereas no significant correlation was observed for left lung V_20_ or Lung-L*_max_* (**Table 3**).

**Table 3:** Correlation between CTV volume and radiation dose parameters for organs at risk.

OAR	Parameter	r	*P*
Heart (cGy)	D*_max_*	0.410**	<0.001
Heart (cGy)	D*_mean_*	0.521**	<0.001
Heart(cc)	V_40_	0.517**	<0.001
Lung-L (cGy)	D*_max_*	0.013	0.899
Lung-L (cGy)	D*_mean_*	0.207*	0.043
Lung-L(cc)	V_20_	-0.085	0.410
CB (cGy)	D*_max_*	0.653**	<0.001
CB (cGy)	D*_mean_*	0.410**	<0.001
CB(cc)	V_10_	0.591**	<0.001

**At the 0.01 level (two-tailed), the correlation is significant. *At the 0.05 level (two-tailed), the correlation is significant.

## Discussion

Following breast-conserving surgery for breast cancer, target field delineation is typically guided by ESTRO recommendations (5, 6), which describe two principal approaches: delineation based on anatomical landmarks or on breast glandular tissue. However, definitive evidence regarding which method provides superior clinical benefit remains limited. In our study, the CTV volume delineated according to mammary gland anatomy was significantly smaller than that defined by broader anatomical structures (432.87 ± 184.09 cc vs. 582.59 ± 232.55 cc, P < 0.001). This reduction in target volume was associated with lower radiation exposure to adjacent organs at risk. Specifically, the left lung V20, mean heart dose, and maximum dose to the contralateral breast were all significantly reduced (P < 0.001). For the heart—an organ in which long-term morbidity is strongly associated with radiation dose (10, 11), —both Dmax and Dmean were significantly lower (P < 0.05). These findings align with the ongoing emphasis on minimizing cardiac and pulmonary exposure in breast cancer radiotherapy, although the dosimetric nature of the study precludes conclusions regarding clinical outcomes.

In radiotherapy, increasing target volume size and irregularity complicates treatment planning and uniform dose distribution. When comparing the two contouring approaches under the requirement that at least 95% of the PTV receives 100% of the prescribed dose, dose extremes within the target were more favorable with mammary gland–based delineation. Compared with anatomical landmark delineation, the glandular group demonstrated significantly lower D*_max_* values (5385.28 ± 41.31 vs. 5407.01 ± 71.20, P = 0.014) and significantly higher D*_min_* values (4289.18 ± 472.86 vs. 4130.09 ± 507.45, P < 0.001). Consequently, the HI was superior in the mammary gland group (1.036 ± 0.01 vs. 1.04 ± 0.01, *P* < 0.001), indicating improved dose uniformity. It is important to emphasize, however, that statistical significance does not necessarily equate to clinical significance. Although several dosimetric parameters demonstrated statistically significant differences, the absolute magnitude of some changes—particularly in CI and HI—was small and unlikely to influence clinical decision-making. These results should therefore be interpreted with caution and within the context of their limited clinical impact.

Because the target volume in breast-conserving surgery is constrained by chest wall curvature, larger CTVs inevitably encompass more lung and heart tissue when tangential irradiation is applied. In the CTV*_gl_* group, radiation doses to the heart were significantly reduced across all parameters—including D*_max_*, D*_mean_*, and V_40_—compared with the CTV*_an_* group. Similar reductions were observed for the left lung and CB. Correlation analysis demonstrated a significant positive relationship between CTV volume and doses to the heart and contralateral breast (all *P* < 0.001). In contrast, no significant correlation was found for the left lung (all *P* > 0.05), except for Lung L*_mean_* (r = 0.207, *P* = 0.043). These findings suggest that left lung dose is influenced by multiple factors, including beam geometry, thoracic anatomy, and target volume shape—and that the weaker correlation with CTV volume may reflect a relatively larger contribution from planning decisions. However, this interpretation requires further investigation. Overall, reductions in CTV volume were consistently associated with lower doses to the heart, left lung, and contralateral breast (**Table 1**).

The reductions in Heart *_mean_*, heart V_40_, and left lung V_20_ observed in the CTV*_gl_* group fall within ranges that have been associated with clinically relevant changes in NTCP in previous studies. However, because the present study did not include NTCP modeling or clinical outcome validation, the clinical implications of these dosimetric improvements remain uncertain. Accordingly, the observed dose reductions should be regarded as hypothesis-generating rather than definitive evidence of clinical benefit.

Systemic therapies for breast cancer are also associated with adverse cardiac effects (9). Given the heart’s low tolerance for radiation, even modest increases in exposure can result in clinically significant damage. Evidence consistently demonstrates a positive correlation between cardiac dose and the incidence of adverse cardiac events (12, 13). Thus, cardiac protection remains a critical priority in radiotherapy planning, particularly for left-sided disease. Although techniques such as deep inspiration breath hold and heart displacement devices have been explored to reduce cardiac exposure (14–16), the achieved reductions in irradiated cardiac volume have been modest (17). Similarly, meta-analyses confirm that radiation pneumonitis risk is significantly correlated with lung V_20_ and mean lung dose (18). While technological advances and equipment innovations continue to improve OAR sparing (2, 3, 19), our findings indicate that differences in CTV delineation are associated with measurable variations in radiation exposure to adjacent organs.

Although the finding that smaller CTV volumes are associated with lower OAR doses is expected, our study provides quantitative evidence of the magnitude of these reductions under two clinically used delineation strategies. However, the oncological implications of reducing CTV volume—such as potential geographic miss or effects on local control—cannot be determined from this dosimetric analysis alone.

In cases where mammary gland tissue is poorly visualized, our center recommends expanding the CTV margin to ensure adequate coverage. Accurate delineation may be complicated by difficulty distinguishing glandular tissue from breast ligaments; therefore, continuous assessment of the glandular layer is essential for precise target definition.

## Limitations

This study has several limitations that warrant consideration. First, although we compared glandular tissue–based and anatomical boundary–based CTV delineation, inter-observer variability in contouring was not systematically assessed, which may influence reproducibility of the dosimetric results. Second, while ongoing advances in radiotherapy equipment and techniques (e.g., IMRT, VMAT, proton therapy) have been shown to improve organ sparing, our analysis did not include a comprehensive comparison across modalities,; therefore, the relative contributions of technology, patient anatomy, and CTV delineation to OAR dose could not be fully distinguished. Third, although significant correlations were observed between CTV volume and doses to the heart and contralateral breast, the statistical models did not account for potential confounders such as patient anatomy, breast size, or beam arrangement, which may influence dose distribution.

In addition, the correlation analyses were subject to statistical dependency because multiple dosimetric endpoints originated from the same patient cohort. As a result, the correlations do not represent independent observations and should be interpreted as exploratory. Furthermore, multiple comparisons were performed without formal adjustment, which may increase the risk of type I error. Importantly, this study was purely dosimetric; no NTCP modeling, toxicity data, or clinical outcome validation was performed. Therefore, the clinical implications of the observed dose differences remain uncertain and cannot be inferred from the present analysis.

Future research should incorporate multicenter cohorts, standardized contouring protocols, and long-term clinical outcome data to validate the dosimetric findings and enhance their translational relevance. In addition, studies with larger sample sizes should employ more advanced statistical approaches—such as multivariate regression or mixed-effects models—to adjust for anatomical confounders and appropriately account for within-subject correlation among dosimetric endpoints.

## Conclusion

This study suggests that CTV delineation is an important factor associated with variations in OAR dose during postoperative breast cancer radiotherapy. Our findings indicate that CTV volume shows the strongest associations with radiation doses to the heart and contralateral breast, whereas left-lung dose appears to be more strongly influenced by additional factors such as beam geometry, thoracic anatomy, and planning decisions. Nevertheless, a consistent reduction in left-lung dose was observed as CTV volume decreased. Smaller CTV volumes inherently reduce the geometric overlap between the target and adjacent organs, thereby lowering the likelihood of high-dose exposure.

Given the established cardiotoxicity of systemic breast cancer therapies, cardiac protection remains a critical priority in radiotherapy planning, particularly following left-sided mastectomy. The present findings underscore the potential value of individualized CTV delineation strategies that aim to balance adequate oncologic coverage with organ preservation. However, these results are purely dosimetric and should not be interpreted as evidence of clinical benefit.

In conclusion, while optimizing CTV delineation may improve dose distribution and reduce exposure to adjacent organs, these dosimetric improvements do not establish reductions in toxicity or improvements in clinical outcomes. Future multicenter studies incorporating standardized contouring protocols, NTCP modeling, and long-term clinical follow-up are needed to validate t these observations and inform clinical practice.

## Data Availability

The original contributions presented in the study are included in the article/supplementary material. Further inquiries can be directed to the corresponding author.

## Glossary

CTV: clinical target volume
OARs: organs at risk
CB: contralateral breast
NTCP: normal tissue complication probability
PTV: planning target volume
D*_min_*: minimum dose
D*_mean_*: maximum dose
SD: Standard deviation
HI: homogeneity index
CI: conformity index
D_98_: The minimum radiation dose delivered to 98% of the target volume
V_40_: The volume of tissue irradiated to 40 Gy
V_20_: The volume of tissue irradiated to 20 Gy
V_10_: The volume of tissue irradiated to 10 Gy
V_5_: The volume of tissue irradiated to 5 Gy
IMRT: Intensity-modulated radiation therapy
CTV*_gl_*: CTV defined according to mammary gland structures
CTV*_an_*: CTV defined according to anatomical landmarks
DVH: dose–volume histogram
Heart*_mean_*: maximum radiation dose to the heart
Heart *_mean_*: mean radiation dose to the heart
Lung-L *_max_*: maximum radiation dose to the left lung
Lung-L*_mean_*: mean radiation dose to the left lung
CB *_max_*: maximum radiation dose to contralateral breast
CB*_mean_*: mean radiation dose to contralateral breast
